# Crossing Borders

**DOI:** 10.5811/westjem.2015.12.29453

**Published:** 2016-01-12

**Authors:** Erik D. Barton

**Affiliations:** University of California Irvine Medical Center, Department of Emergency Medicine, Orange, California

As emergency physicians, we are privileged to be in a field that crosses more boundaries than any other medical specialty. It is a calling. Our skills are portable and transferable across cultural and geographic disparities. For these reasons, many of us are drawn to sharing our knowledge and training across the globe – towards treating patients in underserved and austere environments abroad. The rapid growth of international and global health educational initiatives across our U.S. residency training programs is a direct result of those undeniable forces. Additionally, inclusion of such rotations becomes a powerful resident recruitment tool as more and more of our trainees are looking for these opportunities during their formative years.^1^

However, the survey results reported by Morris et al. in the article “Emergency Medicine Residents Abroad: Current Status and Next Steps” raise some concerns about the initial orientation, mentorship, and preparation practices of our residency programs that offer international rotations.^2^ While we do have many shining examples of institutions that have “all the pieces in place,” these results highlight the need for increased consistency and support practices across all emergency medicine (EM) training programs in sending our residents on global health missions.

Fortunately there are a number of resources available to guide programs in successfully implementing these types of opportunities. Many of our professional societies, including American College of Emergency Physicians,^3^ Emergency Medicine Residents Association,^4^ Society for Academic Emergency Medicine,^5^ and the American Medical Association,^6^ offer comprehensive webpages dedicated to the selection and development of global health rotations for students, residents and fellows. Additionally, many institutional EM residency programs across the country have been offering international rotations for a number of years and have developed extensive websites, protocols, and guidelines based on their experiences. But that is just the start. A number of key components must be present to provide consistency across all programs for the educational benefit and safety of our residents.

First and foremost, there should be a faculty mentor or “champion” in every EM training program for each resident and designated international rotation to coordinate and assist with logistics, planning, and educational goals. These individuals should be uniquely familiar with specific international rotation site(s), having both communicated with key contacts and traveled to the foreign clinics, hospitals, or regions at some point prior to placing residents in those environments. Thus, it is important that department mentors perform an initial site visit to the desired country for a “needs assessment” of the clinical setting(s), educational goals, safety and political stability of the country and region, and support expectations of the hosting entity. This includes identifying an appropriate “supervising physician” onsite if U.S. faculty members will not be traveling directly with trainees (an RRC mandate). The supervising physician is responsible for the educational oversight and post-rotation evaluations of the residents during the entire experience, as well as monitoring their safety and security while staying in the region.

Second, the faculty mentor and their department should establish a “Memorandum of Understanding” (MOU) with the host institution prior to the start of global health rotations. The MOU should clearly specify the roles and responsibilities of the trainees, including duty hours and off-service times. The MOU must also address the insurance coverage and limits of liability for the residents. As suggested by the previous article, up to one third of residents may be sent to locations without specified liability coverage…a very concerning statistic that should not be overlooked. Responsibility for any financial support (travel, food, housing, etc.) should also be clearly outlined in the MOU.

Third, intermittent site visits abroad should be performed by departments and/or mentors on a regular basis (i.e. every 1–2 years) to each of their global health venues. This insures consistency of educational benefits and adherence to Accreditation Council for Graduate Medical Education Core Competencies for resident participants and monitors any potential risks that can arise over time with foreign assignments.

Finally, as suggested in the previous article, establishing a formalized pre-departure training program for residents is crucial. This curriculum could be incorporated into annual residency didactics, provided as additional training sessions, or in conjunction with Global Health Fellowship programs. Understanding culture disparities, travel safety, and regional disease prevalence and patterns are essential preparations for our residents traveling internationally.

The popularity and growth of international experiences is inevitable in our specialty. This is evidenced by the expansion of EM residency rotations and Global Health Fellowship programs. It is in our nature to share our emergency medicine expertise, practice in new environments, and experience foreign cultures. However, based on the results of this article, we have more work to do towards safely sending our residents abroad.
